# Exploring Early Childhood Diet, Stress, Trophic Position and Dietary Protein Quality Using Amino Acid Nitrogen Isotope Compositions of Fingernail Keratin

**DOI:** 10.1002/ajpa.70128

**Published:** 2025-10-08

**Authors:** Hana Salahuddin, Andrea L. Waters‐Rist, Fred J. Longstaffe

**Affiliations:** ^1^ Anthropology, Western University London Canada; ^2^ Earth Sciences, Western University London Canada

**Keywords:** amino acids, breastfeeding and weaning, maternal illness, nitrogen isotopes, pregnancy

## Abstract

**Objectives:**

Evaluate the effectiveness of compound‐specific nitrogen isotope analysis of amino acids (CSIA‐AA) in reconstructing early childhood diets and detecting episodes of stress. Examine (1) proline's potential for identifying breastfeeding and weaning; (2) the influence of physiological and pathological stress on AA *δ* ^15^N; (3) the reliability of trophic position (TP) estimates from phenylalanine (Phe) and glutamate (Glx) *δ* ^15^N during dietary transitions; and (4) mother‐infant trophic enrichment factors (TEF_Glx‐Phe_) as indicators of infant dietary protein quality.

**Materials and Methods:**

Three mother‐infant dyads provided fingernail clippings (*n* = 43) for CSIA‐AA analysis pre‐ and post‐birth, alongside dietary and health surveys.

**Results:**

Proline *δ* ^15^N was elevated by 2.4‰–3.5‰ in exclusively breastfed infants compared to their mothers and decreased by 2.2‰–4.1‰ during weaning. Phenylalanine *δ* ^15^N showed large positive shifts (e.g., by 6.7‰) during maternal stress, despite being a source AA expected to remain stable. TP differences between mother‐infant pairs were minimal (−0.2 to 0.1), except for one pair with higher infant TP (by 0.6–1.5). The calculated TEF_Glx‐Phe_ for infants ranged from −1.4‰ to 11.3‰.

**Discussion:**

Proline *δ* ^15^N reliably tracks nutritional transitions, likely due to its role in arginine synthesis during infancy. The unexpected variability in *δ* ^15^N_Phe_ complicates its use in TP and dietary protein quality assessments. This variability may result from phenylalanine's slow turnover and delayed dietary incorporation during endogenous catabolism. TP is an unreliable marker of breastfeeding or weaning. TEF_Glx‐Phe_ for infants seems indicative of high dietary protein quality, but interpretations must consider the influence of non‐dietary factors on *δ* ^15^N_Phe_.

## 1 Introduction

Nitrogen isotope analysis has become a cornerstone of bioarchaeological research, especially for studying breastfeeding and weaning practices. Such work has become commonplace, as demonstrated by a 2023 publication by Waters‐Rist (2023) that compiled 97 isotopic studies of infant (≤ 11 months) and young child (1–6 years) feeding in pre‐modern populations. Nitrogen isotope analysis offers invaluable insights into early childhood diet and health across diverse cultures and periods—information that is not easily discernible in the archaeological record (e.g., Dupras et al. 2001; Eerkens and Bartelink 2013; Fuller et al. 2006; Greenwald et al. 2016, 2023; Herring et al. 1998; Katzenberg et al. 1993; Katzenberg and Pfeiffer 1995; Mays 2002; Richards et al. 2002; Schurr 1997, 1998; Schurr and Powell 2005; Wright and Schwarcz 1998, 1999; Waters‐Rist et al. 2011, 2022). To expand current applications, our study assesses the efficacy of a more refined technique—compound‐specific nitrogen isotope analysis (CSIA) of amino acids (AAs)—in reconstructing early childhood diets. We analyze fingernail samples from three contemporary mother‐infant pairs to determine new perspectives for bioarchaeological breastfeeding and weaning research. Our first objective is to assess the applicability of the AA proline in determining breastfeeding and weaning periods, contributing to Harris et al.'s (2024) CSIA‐AA infant feeding investigation that used fingernail clippings from one contemporary mother‐infant dyad. Our second objective is to explore the effects of physiological and pathological stress on AA *δ* ^15^N, a topic that has been understudied but has important implications for the accurate application and interpretation of CSIA‐AA data.

A common technique in compound‐specific nitrogen isotope analysis of AAs is the calculation of the trophic position (TP) of consumers using the isotopic spacing between two AAs: glutamic acid (*δ* ^15^N_Glu_) and phenylalanine (*δ* ^15^N_Phe_) (Bradley et al. 2015; Ishikawa et al. 2018; Itahashi et al. 2014; Naito, Bocherens, et al. 2016; Naito, Chikaraishi, et al. 2016). This calculation depends on the universally accepted “trophic enrichment factor” (TEF) value of 7.6‰, which has been applied to various food chains. Recent research, however, has found that variability in TEF across taxa may relate to dietary protein quality (McMahon and McCarthy 2016). As this is yet to be applied to breastfeeding and weaning, our third and fourth objectives are to investigate if this universal TEF is useful in reconstructing dietary transitions in childhood and if the calculated TEF for mother‐infant pairs can inform us about infant dietary protein quality. The investigation of dietary protein quality during early childhood could serve as a novel tool for examining malnutrition, health, and disease in past children.

While *δ* ^15^N results are commonly paired with stable carbon isotope (*δ* ^13^C) analysis, the distinct metabolic pathways of carbon and nitrogen in AAs make such data more comprehensible when considered separately. The *δ* ^13^C AA data from the mother‐infant dyads examined in this study are presented in Salahuddin et al. (2025), showing, for example, that the *δ* ^13^C of glycine, and to a lesser extent glutamate, can be used to identify the onset of exclusive breastfeeding and end of weaning.

### 1.1 Biochemical Mechanisms Underlying *δ* ^15^N Variation in AAs

The *δ* ^15^N of each AA varies based on the number of enzyme‐catalyzed reactions involved in its synthesis. This process includes breaking or forming a C–N bond and acquiring nitrogen from either the metabolic nitrogen pool or directly from the diet (McMahon and McCarthy 2016). Amino acids closely associated with the metabolic nitrogen pool, involved in extensive synthesis reactions, such as glutamic acid (Glu), alanine (Ala), proline (Pro), and valine (Val), are termed trophic amino acids (Tr‐AAs). Specifically, these Tr‐AAs undergo deamination, the removal of an amino group, and transamination, a biochemical reaction transferring an amino group to a keto acid, to generate new AAs. The process of deamination and transamination discriminates against ^15^N when transferring an amino group from a precursor AA (e.g., glutamic acid or glutamine) to create a new AA. Consequently, the newly formed AA is depleted of ^15^N compared to the original AA, contributing to the progressive enrichment of ^15^N in the metabolic nitrogen pool through continued reactions of endogenous AAs (O'Connell 2017). These endogenous AAs are eventually incorporated into tissues, such as fingernails, hair, bone, and teeth.

Amino acids that acquire nitrogen directly from diet, such as phenylalanine (Phe), are termed source amino acids (Src‐AAs). The *δ* ^15^N of consumer Src‐AAs aligns with those of primary producers, as they avoid nitrogen exchange with the metabolic pool and undergo minimal enzyme‐catalyzed reactions (McClelland and Montoya 2002; Naito, Honch, et al. 2010; Naito, Chikaraishi, et al. 2010; O'Connell 2017). Early nitrogen CSIA studies, conducted on invertebrates, suggested that glycine (Gly) and serine (Ser) were also Src‐AAs (McClelland and Montoya 2002; Popp et al. 2007). However, later research on vertebrates and upper trophic species demonstrated marked variability in diet‐tissue offsets for these AAs. It was found that like Tr‐AAs, Ser and Gly can also receive nitrogen from the body's central nitrogen pool, via Ala (McMahon and McCarthy 2016). Threonine (Thr) is unique among the AAs in collagen because it becomes progressively depleted of ^15^N as it moves up the food chain. Furthermore, *δ* ^15^N_Thr_ has been shown to be negatively correlated with the quantity of dietary protein (Fuller and Petzke 2017). Hence, it is categorized as a “metabolic” AA, but as O'Connell (2017) notes, all AAs are metabolic.

In addition to metabolic differences in AAs, pathological stressors and physiological processes such as starvation or gestation can influence the isotopic composition of tissues and their constituent AAs (Fuller et al. 2004, 2005; Hobson et al. 1993). Some studies have shown that fasting induces a slight but measurable average increase of +0.5‰ ± 0.2‰ in an organism's *δ* ^15^N, with tissue type as the only significant moderator (McCue 2007; Hertz et al. 2015; although see Kempster et al. (2007) for a study that did not find an isotopic change). During fasting, organisms “consume” themselves by first breaking down lipid‐rich (adipose) tissues and then protein‐rich (skeletal muscle) tissues for metabolism (Fuller et al. 2005). Endogenous AAs, which are ^15^N‐enriched relative to dietary AAs, are broken down for glucose synthesis through gluconeogenesis, leading to an additional increase in *δ* ^15^N of the remaining AAs in the body's pool, eventually used for tissue synthesis. Trophic AAs, such as glutamic acid, linked to nitrogen cycling, undergo more deamination during stress or fasting (O'Connell 2017). In contrast, Src‐AAs experience minimal to no *δ* ^15^N change, as they are not closely associated with nitrogen cycling and cannot be easily deaminated without catabolizing the entire compound (O'Connell 2017). Based on these distinctions, the analysis of the *δ* ^15^N of AAs may offer insights into biochemical mechanisms that change due to pathological or physiological factors (Ohkouchi et al. 2017; Whiteman et al. 2019).

### 1.2 Trophic Enrichment Factor

The identification of Src‐ and Tr‐AAs prompted the development of indices that could be used to identify trophic positions (TP) within and across food webs despite background variation in isotopic values (e.g., Chikaraishi et al. 2009, 2014; McCarthy et al. 2007; Popp et al. 2007). Arguably, the most applied and accurate of these indices (Δ^15^N_Glu‐Phe_) was developed by Chikaraishi et al. (2009):

$${\mathrm{TP}}_{\mathrm{AA}}=\left[\Delta^{15}N_{\mathrm{Glu}-\mathrm{Phe}}-\beta \right]/\mathrm{TEF}+1$$

TP_AA_ = trophic position calculated from AA isotope analyses, Δ^15^N_Glu‐Phe_ = difference between consumer *δ* ^15^N_Glu_ and *δ* ^15^N_Phe_, β = primary producers Δ^15^N_Glu‐Phe_, 8.4‰ for C_3_ systems (Chikaraishi et al. 2009), TEF = trophic enrichment factor, also known as trophic discrimination factor (TDF).

The legitimacy of the trophic position calculated using this equation is dependent on the TEF. The TEF is calculated by subtracting the ^15^N enrichment (the increase of *δ* ^15^N_Phe_ from diet to consumer) of phenylalanine (Δ^15^N_Phe_) from the ^15^N enrichment of glutamic acid (Δ^15^N_Glu_) (Chikaraishi et al. 2009). Chikaraishi et al. (2007) proposed a TEF_Glu‐Phe_ of 7.6‰, which became widely accepted as a canonical reference for calculating TP using CSIA‐AA across diverse taxa and environments (Choy et al. 2012; Dale et al. 2011; Lorrain et al. 2009, 2015; Miller et al. 2013; Nakatomi et al. 2014). However, variability in TEFs has been noted in several studies, which have been partially attributed to differences in dietary protein quality and quantity (e.g., Hobson et al. 1993; Robbins et al. 2005; Tsahar et al. 2008).

A high‐quality protein diet contains AA compositions similar to the consumer's needs, while a low‐quality protein diet requires reliance on endogenous sources to meet AA requirements. Based on McMahon and McCarthy's (2016) meta‐analysis of controlled feeding experiments, organisms consuming high‐quality protein had significantly lower TEF_Glu‐Phe_ values than low‐quality protein consumers. This difference occurs because organisms with a low‐quality protein diet must obtain a large portion of their nitrogenous compounds from endogenous sources that have already been enriched in ^15^N relative to the dietary AAs, in other words, from recycled bodily tissues. In contrast, organisms that feed on high‐quality protein diets can satisfy their AA requirements from direct routing without transamination and deamination (Ambrose and Norr 1993; Schwarcz and Schoeninger 1991), meaning that consumption of higher‐quality diets should lead to lower enrichment of ^15^N in Tr‐AAs.

Typically, breastmilk contains high concentrations of the AAs glutamate, proline, and leucine; hence a breastfeeding infant's TEF_Glu‐Phe_ should be smaller than 7.6‰ as glutamate can be directly routed from diet (Davis et al. 1994). Depending on the protein quality of the infant's weaning diet, however, the TEF_Glu‐Phe_ may increase or decrease, which could alter dietary interpretations. Maternal diet and geographic and/or population genotypic variation have been found to have little effect on the AA composition of breastmilk (Garcia‐Rodenas et al. 2016; Zhang et al. 2013); accordingly, a change in an infant's TEF_Glu‐Phe_ should only arise as a result of changes in their diet. This phenomenon has not been previously explored for infants and young children; hence the present work comprises a formative study for future research investigating childhood diet using CSIA‐AA.

## 2 Materials and Methods

### 2.1 Sample Collection

Ethics approval was obtained from the Western University Non‐Medical Research Ethics Board (NMREB) (Supporting Information 1). Participants were asked to complete dietary and health surveys for themselves and their infant (see Supporting Information 2). Fingernails were chosen for analysis because they grow incrementally and have the potential to provide longitudinal dietary information if collected over time (e.g., Buchardt et al. 2007; Fraser et al. 2006; O'Connell et al. 2001; Williams and Katzenberg 2012). A total of 43 fingernail samples were collected from three mother‐infant dyads both before and after birth, continuing until the infants reached at least 6 months of age (Supporting Information 3, Table S1). Participants were recruited through word‐of‐mouth. The study occurred during the COVID‐19 pandemic, which presented challenges to recruitment, sample collection, and laboratory use. Given these challenges, we focused on three dyads for feasibility. Mothers were recruited based on their willingness to exclusively breastfeed for part of infancy and their ability to provide at least one sample before birth for comparison. No other restrictions were imposed, allowing for the observation of diverse feeding practices. Two dyads resided in Canada (MOM‐CHIL 1 and 3), while the third was located in Western Europe (MOM‐CHIL 2). We acknowledge that this study is potentially limited by selection bias, which may result in participants with shared socioeconomic or cultural backgrounds, and by a small sample size, which reduces generalizability. Individual feeding variation could also obscure broader trends. However, since our goal was to test whether CSIA‐AA can detect dietary changes over time, the specific feeding practices of participants are less central to our aims.

All three dyads followed an omnivorous diet. MOMs 1 and 2 exclusively breastfed for 4 months before introducing solids and gradually weaning. MOM 1 supplemented with formula from 4 to 12 months and ceased breastfeeding by 9 months, while MOM 2 fully weaned by 12 months. MOM 3 initially mixed formula and breastmilk due to early feeding challenges (see Supporting Information 2) but exclusively breastfed from 8 weeks to 4 months. Solids were introduced at 4 months, with breastfeeding tapering off by 7 months and ceasing by 12 months.

### 2.2 CSIA‐AA

Fingernail debris was scraped off using a scalpel and then samples (6 mg) were degreased as per the protocol established by Tea et al. (2013). This involved an ethyl acetate wash followed by a hexane wash.

CSIA‐AA preparation involved a combination of protocols from Corr et al. (2007a, 2007b), Styring et al. (2010), and Schwartz‐Narbonne et al. (2015) (see Supporting Information 4). For the analysis, samples were dissolved in ethyl acetate (1 mL) and injected twice into the Thermo Trace GC 1310 gas chromatograph coupled to a Thermo Scientific Delta V Advantage IRMS via a GC Iso‐Link II Combustion interface. A standard AA mixture of alanine, valine, proline, glutamate, and phenylalanine (purchased through Sigma–Aldrich, UK, derivatized in‐house) was analyzed after every 3rd to 6th sample injection, and the averages from the standard mixture were used for the calibration of the samples. The measurements are reported in per mil (‰) and calibrated to AIR. The precision of AA *δ* ^15^N was < 1‰ in the standard mixture (3–6 times per sequence) across the analytical sessions (*n* = 15), and sample duplicates differed by ±0.5‰.

### 2.3 Statistical Analyses

Linear mixed‐effects models (LMMs) were used to assess the effects of feeding mode, maternal status (i.e., mother vs. infant), and stress exposure on *δ* ^15^N_AA_, with Individual ID as a random intercept. Models were run using the *lmer()* function (lme4 package, R), with fixed effects for feeding mode, status, and stress. Predictions with 95% confidence intervals were generated using *ggeffects* and visualized with *ggplot2*. Feeding mode was treated as an ordered factor to reflect chronological dietary transitions.

## 3 Results

We were able to retrieve *δ* ^15^N_AA_ data for all samples, except MYN2 and MYN5 belonging to MOM 1 (see Table S1 in Supporting Information 5). Baseline resolution, which is essential for the determination of reliable *δ* ^15^N_AA_ values, was obtained for eight AAs. Assessment of sample molecular preservation is detailed in Supporting Information 4. Based on the AA percentages derived from individual peak areas obtained during gas chromatography with flame ionization detection (GC‐FID), the molecular preservation of nail keratin samples was deemed sufficient to proceed. Nitrogen isotope profiles were created for all participants using the *δ* ^15^N_AA_ data. Individual profiles with all 8 AAs are available in Supporting Information 5 (Figures S1–S6). Nitrogen isotope profiles with select AAs are presented below to highlight the differences in Src‐ and Tr‐AA patterning for each individual.

### 3.1 Breastfeeding and Weaning— *δ* ^15^N of Proline

The mean nitrogen isotope compositions of AAs reveal that the largest differences between mothers and their infants occurred for glycine (+1.9‰), valine (+1.9‰), and proline (+1.7‰), while the smallest differences were measured for threonine (−0.1‰) and glutamate (−0.3‰) (see Table S2 in Supporting Information 5). A comparison of the *δ* ^15^N_AA_ profiles of mothers and their infants revealed that proline was the most reliable indicator of exclusive breastfeeding and weaning.

#### 3.1.1 MOM‐CHIL 1

**FIGURE 1:**
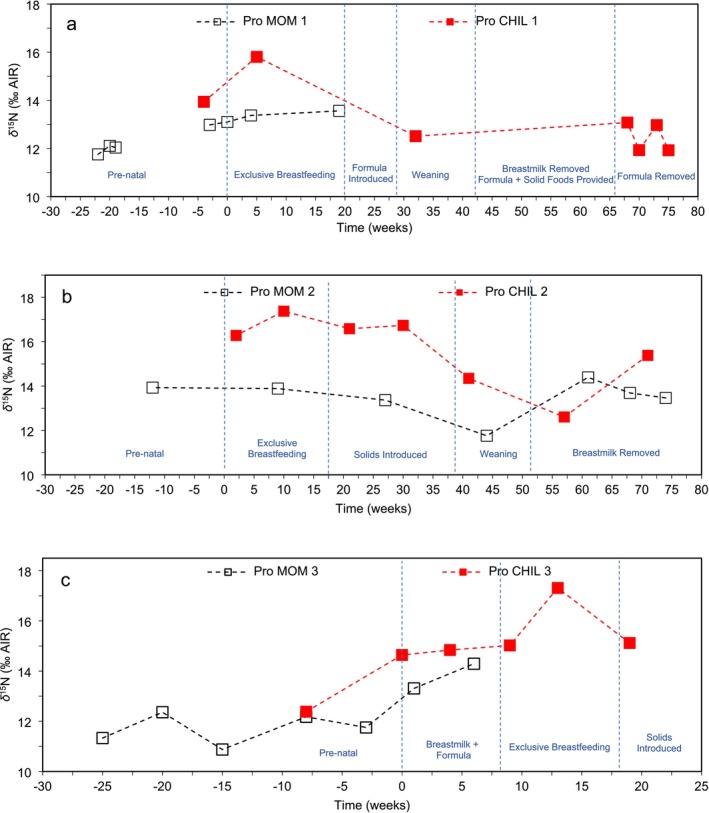
Nitrogen isotope profiles of proline (Tr‐AA) for mother (black open squares) and child (red solid squares) dyads. (a) MOM and CHIL 1 from 22 weeks before to 75 weeks after parturition, (b) MOM and CHIL 2 from 12 weeks before to 74 weeks after parturition, and (c) MOM and CHIL 3 from 25 weeks before to 19 weeks after parturition. Fingernail keratin time of formation error margins are 4 weeks for mothers and 2 weeks for infants and δ^15^N_Pro_ analytical error is ±0.5‰. These error margins are not shown on figure to improve trend visualization.

In Figure 1a, CHIL 1's *δ* ^15^N_Pro_ shows a 2.4‰ increase compared to the mother during the exclusive breastfeeding period and a subsequent 1.1‰ decline during the weaning phase. These fluctuations in proline are consistent with the reported ~3‰ increase and subsequent decrease observed in bulk tissue *δ* ^15^N studies of infant diet (Fogel 1989; Fuller et al. 2006).

#### 3.1.2 MOM‐CHIL 2

During exclusive breastfeeding, the *δ* ^15^N_Pro_ of CHIL 2 was 2.4‰–3.5‰ higher than that of the mother (Figure 1b). Over the course of the gradual weaning process, the infant's *δ* ^15^N_Pro_ decreased by 4.1‰. The final data point for CHIL 2 *δ* ^15^N_Pro_, formed at 74 weeks, reflects the diet following the complete removal of breastmilk.

#### 3.1.3 MOM‐CHIL 3

Figure 1c illustrates fluctuations in MOM 3's *δ* ^15^N_Pro_, showing variation of ~1‰ during the prenatal period. A subsequent increase of 2.5‰ occurred from 3 weeks before birth and onwards. Similarly, CHIL 3's *δ* ^15^N_Pro_ increased by 2.3‰ between 3 weeks before and the week of birth. Due to low breastmilk supply, CHIL 3 was supplemented with formula until ~8 weeks of age when MOM 3 transitioned to exclusive breastfeeding. Between 9 and 13 weeks, during exclusive breastfeeding, CHIL 3's *δ* ^15^N_Pro_ increased by 2.3‰ thus closely matching the exclusive breastfeeding values of the other two infants. After 13 weeks, *δ* ^15^N_Pro_ declined by 2.2‰, corresponding to the introduction of solid foods.

**FIGURE 2:**
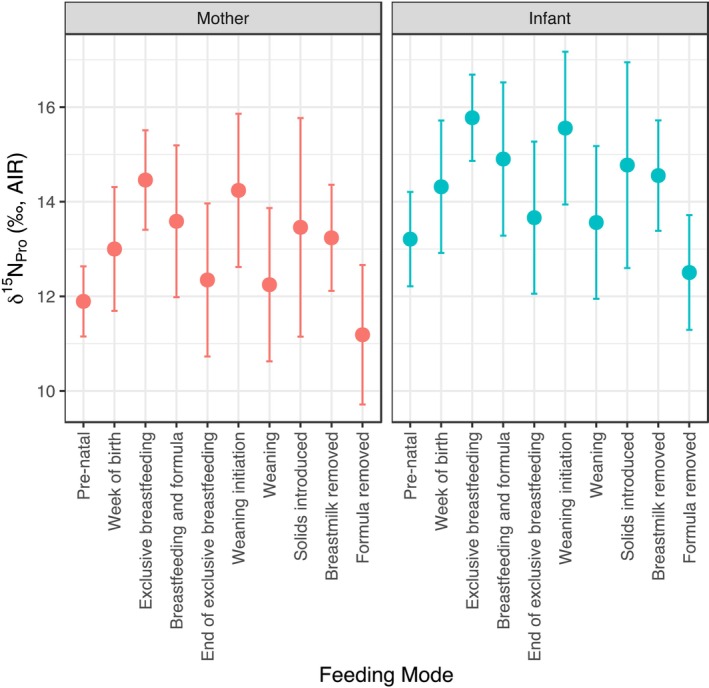
Model‐predicted *δ* ^15^N_Pro_ across feeding stages for mothers (*n* = 3) and infants (*n* = 3) with 95% confidence intervals, obtained using a linear mixed‐effects model (*δ* ^15^N_Pro_~feeding mode + maternal status + (1|individual ID)). Feeding stages are ordered chronologically from gestation through post‐weaning transitions.

A linear mixed‐effects model with Individual ID as a random intercept examined feeding mode and maternal status effects on *δ* ^15^N_Pro_ (Figure 2). The model‐predicted results showed *δ* ^15^N_Pro_ increasing by up to 2.6‰ during exclusive breastfeeding and staying elevated through early weaning (e.g., +2.4‰ at weaning start). Additionally, infants' *δ* ^15^N_Pro_ was significantly higher than mothers' by 1.3‰ (SE = 0.4‰, *t* = 2.8).

### 3.2 Non‐Dietary Changes— *δ* ^15^N of Phenylalanine

**FIGURE 3:**
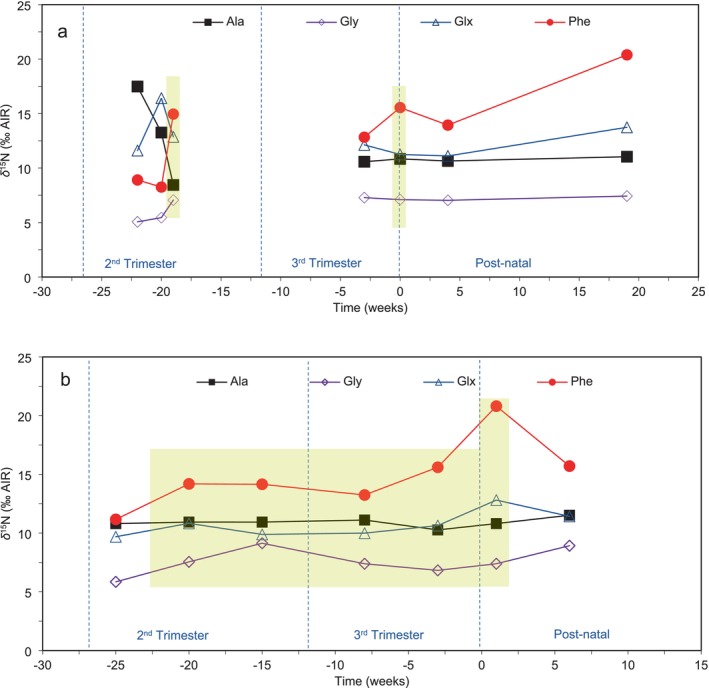
Nitrogen isotope data for the Tr‐AAs alanine (squares), glycine (rhombi), and glutamate (triangles), and the Src‐AA phenylalanine (circles) for (a) MOM 1 from 22 weeks before to 19 weeks after parturition, and (b) MOM 3 from 25 weeks prior to 6 weeks after parturition. Yellow boxes indicate periods of stress. Fingernail keratin time of formation error margins are 4 weeks for mothers and *δ* ^15^N_Pro_ analytical error is ±0.5‰. Errors margins are not shown on the figure to improve trend visualization.

In contrast to the expectation that Tr‐AAs would exhibit elevated *δ* ^15^N during periods of stress, our study found that the Src‐AA, phenylalanine, demonstrated greater enrichment in ^15^N during pathological or physiological stress. Figure 3a illustrates MOM 1's *δ* ^15^N profile for the Tr‐AAs, alanine, glycine, glutamate, as well as the Src‐AA, phenylalanine. From 20 to 19 weeks before birth, the *δ* ^15^N of glycine and phenylalanine rose by 1.6‰ and 6.7‰, respectively, while the *δ* ^15^N of alanine and glutamate declined by 4.8‰ and 3.5‰. These fluctuations coincide with the period (23rd week of pregnancy in the 2nd trimester) when MOM 1 had COVID‐19. From this period onwards, the *δ* ^15^N of all AAs, except phenylalanine and glutamate, remained relatively stable. During the week of delivery, *δ* ^15^N_Phe_ increased by 2.7‰, while *δ* ^15^N_Glx_ declined by 0.9‰, in comparison to values 3 weeks prior, possibly due to the physiological strain of giving birth.

MOM 3 experienced multiple health complications during pregnancy. In the second trimester, she suffered from leg and foot edema, migraines, and fatigue. In the third trimester, she developed respiratory syncytial virus (RSV), which progressed to pulmonary pneumonia, along with severe anemia. Additionally, a diagnosis of preeclampsia led to labor induction 3 weeks before her due date. Similar to MOM 1, MOM 3's *δ* ^15^N profile of phenylalanine, glycine, and glutamate (Figure 3b) demonstrates an increase of 3.0‰, 1.7‰, and 1.1‰, respectively, during the second trimester, between 25 and 20 weeks before birth. Nitrogen isotope compositions continued to increase for phenylalanine and glutamate by 1.5‰ and 0.8‰, respectively, into the third trimester, while the *δ* ^15^N of glycine decreased by 2.3‰. After birth, between 1 and 6 weeks, *δ* ^15^N for phenylalanine and glutamate decreased by 5.1‰ and 1.4‰, and *δ* ^15^N_Gly_ increased by 1.5‰. Alanine *δ* ^15^N remained stable during both the prenatal and postnatal period. This tells us that the *δ* ^15^N of some Src‐AA (phenylalanine) and Tr‐AAs (glutamate and glycine) were altered by pathological stress.

**FIGURE 4:**
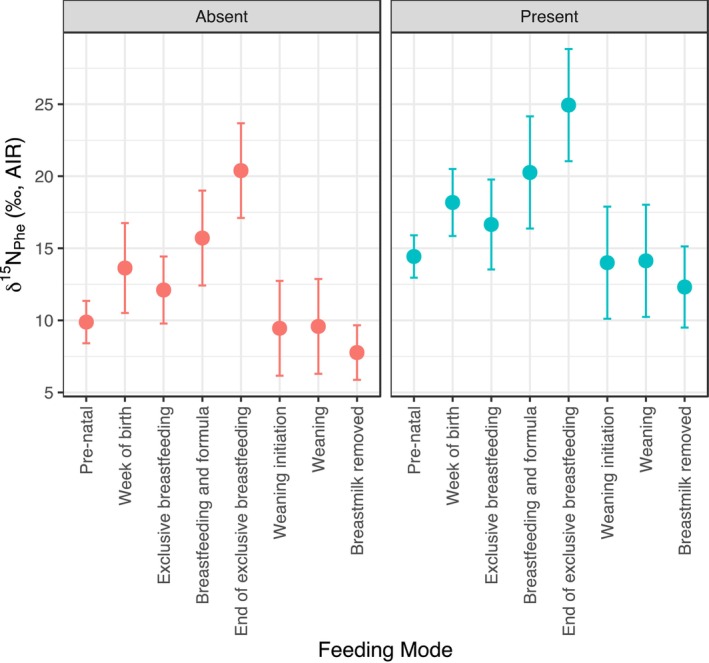
Model‐predicted *δ* ^15^N_Phe_ for mothers (*n* = 3) with “absent” or “present” self‐reported stress, plotted across pre‐natal and infant feeding stages with 95% confidence intervals, obtained using a linear mixed‐effects model (*δ* ^15^N_Phe_~gestation + PostNatal + StressEvent + (1|Individual.ID)).

A linear mixed‐effects model assessed the impact of feeding stage and maternal stress on *δ* ^15^N_Phe_, with random intercepts for Individual ID. As shown in Figure 4, in the presence of stress events, *δ* ^15^N_Phe_ was predicted to increase by 4.6‰ in mothers (SE = 0.9‰, *t* = 4.9), indicating a strong effect regardless of feeding stage. The model also predicted that for all mothers, *δ* ^15^N_Phe_ rose at birth by 3.8‰ (SE = 1.2‰, *t* = 3.0) and was 10.5‰ higher at the end of exclusive breastfeeding (SE = 1.6‰, *t* = 6.5) than during other stages. Levels declined or stabilized during weaning and post‐weaning stages (e.g., post‐weaning, *β* = −2.1‰, SE = 1.1‰, *t* = −2.0), consistent with reduced metabolic demands.

### 3.3 Trophic Positioning of Mother—Infant Pairs

The trophic position for each sample was calculated using Chikaraishi et al.'s (2014) equation with the TEF of 7.6‰ (Table 1). Generally, the TP differences between mother‐infant pairs were negligible (ranging from −0.2 to 0.1) across all feeding stages (i.e., pre‐natal, breastfeeding, and weaning), with the exception of MOM‐CHIL 1. During exclusive breastfeeding, CHIL 1's TP was 1.5 higher than the mother's, and at the end of exclusive breastfeeding, it was 1.3 higher. Similarly, during the pre‐natal period, CHIL 1's TP was 0.6 higher than the mother.

**TABLE 1:** Comparison of trophic positions (TP) between mothers and infants from pre‐ and postnatal time periods. Trophic position was calculated using Chikaraishi et al.'s (2014) equation and the TEF value of 7.6‰.

Pre‐natal and post‐natal stages	Time of sample formation (weeks; ±4 weeks)	TP	Time of sample formation (weeks; ±2 weeks)	TP	TP difference between mother and child
*PAIR 1*	*MOM 1*		*CHIL 1*		
Pre‐natal	−3	2.0	−4	2.6	0.6
Exclusive breastfeeding	4	1.7	5	3.2	1.5
End of exclusive breastfeeding	19	1.2	32	2.5	1.3
*PAIR 2*	*MOM 2*		*CHIL 2*		
Exclusive breastfeeding	9	2.3	10	2.1	−0.2
Weaning initiation	27	2.4	30	2.6	0.2
Middle of weaning	44	2.3	41	2.4	0.1
Weaning completion	61	2.5	57	2.3	−0.2
Post‐weaning	74	2.9	71	2.2	−0.7
*PAIR 3*	*MOM 3*		*CHIL 3*		
Pre‐natal	−8	1.7	−8	1.5	−0.2
Week of birth	1	1.1	0	1.3	0.2
Breastfeeding and formula	6	1.5	4	1.6	0.1

Along with calculating the TP of individuals, we also calculated the TEF_Glx‐Phe_ for each pair during the pre‐ and post‐natal stages to see how it compared to the universally accepted value of 7.6‰. The TEF_Glx‐Phe_ for mother‐infant pairs varied from −1.4‰ to 11.3‰ (Table 2). In MOM‐CHIL pairs 2 and 3, the difference never exceeded 2.0‰, clearly well below the standard TEF_Glx‐Phe_ of 7.6‰. Recall that lower TEF_Glx‐Phe_ values are indicative of a high‐quality protein diet, whereas higher TEF_Glx‐Phe_ values suggest a low‐quality protein diet (McMahon and McCarthy 2016). MOM‐CHIL pair 1 had markedly different TEF_Glx‐Phe_ values of 4.5‰ (prenatal), 11.3‰ (exclusive breastfeeding), and 9.8‰ (weaning). This variation may be attributed to changes in the mother's diet between the prenatal and postnatal periods, a possibility further explored in the discussion.

**TABLE 2:** The TEF_Glx‐Phe_ for mother‐infant pairs during phases when infants were completely (e.g., prenatal, breastfeeding) or heavily (e.g., during weaning) dependent on maternal resources for nutrition.

Pre‐natal and post‐natal stages	Time of sample formation MOM (weeks; ±4 weeks)	Time of sample formation CHIL (weeks; ±2 weeks)	TEF_Glx‐Phe_ (‰)
Pair 1			
Pre‐natal	−3	−4	4.5
Exclusive breastfeeding	4	5	11.3
End of exclusive breastfeeding	19	32	9.8
Pair 2			
Exclusive breastfeeding	9	10	−1.2
Weaning initiation	27	30	2.0
Middle of weaning	44	41	1.0
Pair 3			
Pre‐natal	−8	−8	−1.0
Week of birth	1	0	2.0
Breastfeeding and formula	6	4	0.5

## 4 Discussion

### 4.1 Reconstructing Breastfeeding and Weaning

Harris et al. (2024) were the first to publish nitrogen isotope results for a long‐term CSIA‐AA study of mother‐infant fingernail clippings. Their study revealed that proline and threonine were effective tracers of human milk in the infant's diet, as the isotopic compositions of these AAs remained distinct from maternal isotope compositions until breastfeeding ceased. Similarly, *δ* ^15^N_Pro_ in our study clearly demonstrated exclusive breastfeeding and weaning for all three infants (refer to Figure 1). Throughout exclusive breastfeeding, infant *δ* ^15^N_Pro_ consistently was 2.4‰–3.5‰ higher than maternal values, aligning with the expected range (2‰–3‰) of increase in *δ* ^15^N_bulk_ observed by previous researchers for breastfeeding infants (Fogel 1989; Fuller et al. 2006). During weaning, CHIL 1 and CHIL 2 experienced decreases of 3.3‰ and 4.1‰ in *δ* ^15^N_Pro_, respectively, while CHIL 3's *δ* ^15^N_Pro_ decreased by 2.2‰ following the introduction of solid foods. These patterns were supported by LMM predictions, which showed significant effects of feeding mode on *δ* ^15^N_Pro_, with infants exhibiting consistently higher values than mothers during exclusive breastfeeding, followed by a decline across weaning stages (Figure 2).

Additionally, the average difference in *δ* ^15^N_Pro_ between mothers and their infants, accounting for both prenatal and postnatal periods (+1.7‰), is one of the highest compared to other AAs. This finding aligns with Romek et al.'s (2013) study, which recorded the highest difference in *δ* ^15^N_Pro_ (+3.3‰) between milk proteins from mothers and hair proteins from their infants. This ^15^N enrichment in proline may be linked to its role in arginine synthesis in infants (Harris 2020; Harris et al. 2024). Considering the low arginine content in breastmilk, a high rate of arginine synthesis is implied for protein synthesis (Svanberg et al. 1977). Tomlinson et al.'s (2011) multi‐tracer isotope study of enterally fed neonates found that proline is a major contributor to arginine synthesis in pre‐term infants. *De novo* arginine synthesis provides more arginine than is derived from breastmilk, leading to substantial catabolism of proline and subsequent ^15^N enrichment of proline (Tomlinson et al. 2011). Thus, the consistent and notable increase in *δ* ^15^N_Pro_ supports the effective utilization of *δ* ^15^N_Pro_ in reconstructing breastfeeding and weaning practices.

### 4.2 Catabolism and Its Influence on the *δ* ^15^N of AAs

To date, only a limited number of studies have investigated the impact of catabolism on the *δ* ^15^N_AA_ of human tissues (e.g., Krishnamurthy et al. 2017). Typically, based on the anticipated metabolic pathways, it is expected that Tr‐AAs, not Src‐AAs, would exhibit modified *δ* ^15^N during periods of stress. However, unexpectedly, the *δ* ^15^N of phenylalanine proved to be the most responsive to stress relative to other AAs. MOM 1's *δ* ^15^N_Phe_ increased by 6.7‰ during the 23rd week of her pregnancy, aligning with COVID‐19 and symptoms such as fatigue and shortness of breath. MOM 1's *δ* ^15^N_Phe_ also increased by 2.7‰ during the week of birth. Interestingly, MOM 1 reported that this birth was much more difficult than her previous one. Similarly, MOM 3 experienced a 3.0‰ increase in *δ* ^15^N_Phe_ at the beginning of her second trimester (25–20 weeks before delivery), coinciding with symptoms including leg and feet edema, migraines, and fatigue. From 8 weeks before parturition (the start of the third trimester) until the first week after birth, MOM 3 exhibited a 7.6‰ increase in *δ* ^15^N_Phe_. During the third trimester, MOM 3 faced numerous serious health issues and due to complications from preeclampsia, she underwent induction 3 weeks before her due date. After delivery, she received multiple medical treatments, including two different blood pressure medications, iron supplements, antibiotics for an ear infection, a puffer to alleviate pneumonia, and pain medications due to an emergency C‐section. Based on the profiles of MOM 1 and MOM 3, we predict that phenylalanine *δ* ^15^N can be used to identify periods of stress.

Predictions from the linear mixed‐effects model also revealed clear variation in maternal *δ* ^15^N_Phe_ across infant feeding stages, with the highest values observed at the end of exclusive breastfeeding. This trend, along with elevated *δ* ^15^N_Phe_ throughout several feeding stages in mothers who experienced stress, likely suggests that both the energetic costs of milk production and the additional strain of pathological stress can significantly alter maternal nitrogen metabolism, leaving detectable isotopic signatures.

Our findings are supported by Lübcker et al.'s (2020) CSIA‐AA study of whisker keratin from free‐ranging subadult and adult southern elephant seals, which also examined the effects of catabolism on *δ* ^15^N_AA_. These authors found that *δ* ^15^N of the Src‐AAs phenylalanine, lysine, and tyrosine increased by ~2‰ to 4‰ during fasting in both pups and adult females, suggesting that these AAs were also catabolized to fuel gluconeogenesis. Identifying the precise pathways behind the pronounced increases in *δ* ^15^N_Phe_ during periods of physiological or pathological stress remains challenging. However, Lübcker et al. (2020) propose that the distinct turnover rates of Src‐ and Tr‐AAs may influence the measured isotopic compositions. Downs et al.'s (2014) research on AA turnover rates, or the rate at which AAs are incorporated into proteins, in Pacific white shrimp revealed considerable variation in turnover times among different AAs. Glycine and proline exhibited particularly rapid turnover, whereas glutamic acid had one of the slower rates, with phenylalanine's turnover time being slightly faster than that of glutamic acid. This suggests that once phenylalanine is catabolized to fuel gluconeogenesis, its slower turnover rate delays the incorporation of exogenous or dietary phenylalanine. As a result, the endogenous phenylalanine pool becomes increasingly enriched in ^15^N, leading to elevated *δ* ^15^N_Phe_.

Unsurprisingly then, despite the slow turnover rate of glutamate (a Tr‐AA), it showed inconsistent changes during periods of physiological or pathological stress. This is likely due to its extensive nitrogen exchange with other AAs, as well as the fact that glutamate and glutamine represent the largest nitrogen pool in the body (O'Connell 2017). For example, during the week that MOM 1 had COVID‐19, *δ* ^15^N_Glx_ decreased by 3.5‰, while for MOM 3, it increased by 2.2‰ from 3 weeks before to the week of birth. While our study observed subtle changes in *δ* ^15^N_Glx_, Lübcker et al. (2020) found no distinction in whisker *δ* ^15^N_Glx_ between fasting and foraging in both juveniles and adults, supporting that glutamate is likely not as affected by physiological or pathological stress.

The nitrogen isotope compositions of glycine, a Tr‐AA, exhibited slight elevation during periods of stress. For instance, MOM 1's *δ* ^15^N_Gly_ increased by 1.6‰ during the week she contracted COVID‐19 but remained stable during the week of delivery. Similarly, MOM 3's *δ* ^15^N_Gly_ increased by 3.3‰ over the second trimester but decreased by 2.3‰ over the third trimester before increasing again by 2.1‰ after delivery. Lübcker et al. (2020) similarly observed *δ* ^15^N increase in the Tr‐AAs glycine and serine, which rose by 6.3‰ in subadult seals during fasting compared to foraging. These AAs serve as primary substrates for gluconeogenesis, a crucial biochemical pathway through which dietary or endogenous non‐carbohydrate components (lipids or protein) are converted into glucose (Krebs 1964). Lübcker et al. (2020) suggest that after peptides from the diet are broken down into their constituent AAs, the AAs containing isotopically light (^14^N) amine groups are preferentially deaminated in the first step of gluconeogenesis, resulting in the isotopic enrichment of ^15^N in the remaining free AAs. These remaining AAs are eventually used to synthesize whiskers during fasting when there is no access to exogenous sources, causing the elevated *δ* ^15^N.

Lübcker et al. (2020) also documented a reduction in *δ* ^15^N_Ala_ of ~2‰ to 3‰ in both juvenile and adult females during fasting. They attribute this phenomenon to the glucose‐alanine cycle (Cahill et al. 1981), which serves as the predominant metabolic pathway for nitrogen transfer from muscle to the liver during fasting. This lowering of *δ* ^15^N_Ala_ is theorized to occur through transamination, where ^14^N atoms from glutamate are transferred to alanine. The resultant ^14^N‐rich alanine is transported via blood plasma to the liver. The ^14^N‐rich alanine can also be utilized in the bloodstream to sustain actively growing tissues, such as whiskers, during fasting (Felig et al. 1970; Krebs 1964). In the case of MOM 1 during COVID‐19, it is plausible that pathological, stress‐induced catabolism prompted alanine synthesis through the utilization of the Cahill cycle. However, no significant changes were observed for MOM 3, indicating that further investigation is needed to clarify when and how the Cahill cycle influences *δ* ^15^N_Ala_.

Overall, our findings challenge conventional assumptions regarding the distinct roles of Src‐ and Tr‐AAs under physiological and pathological stress. Notably, phenylalanine exhibited the most substantial change, underscoring its potential as a sensitive marker of stress. However, given the complexities of AA metabolic pathways, further research is essential to understand the underlying mechanisms influencing *δ* ^15^N_AA_.

### 4.3 Trophic Positioning of Mothers and Infants

Our results indicate that the TP of infants cannot serve as a reliable signal for breastfeeding and weaning when compared to the TP of the mother (refer to Table 1). This observation contrasts with the findings of Cheung et al. (2022), who also employed CSIA‐AA to analyze weaning practices in two Middle Neolithic communities in the Paris Basin region. In Cheung et al. (2022), a noticeable breastfeeding signal was observed based on the TP of adults (2.9–3.4) and subadults (3.2–3.7). While differences in TP estimates between Cheung et al. (2022) and our study likely reflect greater consumption of higher‐trophic level foods (e.g., freshwater protein) by Neolithic individuals in the Paris Basin, both studies observed similar TP offsets between mothers and infants. In our study, TP differences ranged from −0.2 to +0.6 (excluding two outliers from MOM‐CHIL 1 during exclusive breastfeeding), while Cheung et al. (2022) reported offsets of +0.3 to +0.8 between adults and subadults. These unexpectedly low values challenge the expectation that exclusively breastfed infants would be one trophic position higher than their mothers, pointing to potential metabolic variations that require further study.

A potential explanation for the limited rise in the TP between mothers and infants could be physiological or pathological conditions affecting *δ* ^15^N_Phe_. Previous research has relied on the *δ* ^15^N difference between glutamic acid (trophic) and phenylalanine (source) to infer the TP of various organisms (e.g., Chikaraishi et al. 2009; Germain et al. 2013; McMahon and McCarthy 2016), assuming that *δ* ^15^N_Phe_ is insensitive to TP and physiology (Chikaraishi et al. 2009). However, our study indicates that non‐dietary factors can cause ^15^N enrichment in phenylalanine and strongly influence TP estimates. Lübcker et al. (2020) also noted that elephant seal pups were about half of a trophic level lower than their mothers, rather than a trophic level higher, due to fluctuations in phenylalanine *δ* ^15^N during fasting. This observation suggests that physiological status must be taken into account when interpreting offsets in AA *δ* ^15^N as proxies for trophic level (Chikaraishi et al. 2009).

While MOM‐CHIL 1 may illustrate a case where TP estimates can help identify breastfeeding and weaning periods, the observed pattern could also result from changes in the mother's diet rather than the child's, as discussed in the following section. Further research with larger sample sizes is needed to determine the reliability of TP estimates for detecting breastfeeding and weaning transitions.

### 4.4 TEF and Dietary Protein Quality

Our findings indicate that, overall, infants consumed high‐quality protein diets, as reflected by low TEF_Glx‐Phe_ values (< 7.6‰) (Table 2). This result aligns with expectations, as breastmilk is known to contain high concentrations of glutamate, proline, and leucine, leading to low TEF_Glx‐Phe_ (Davis et al. 1994). CHIL 1 had higher TEF_Glx‐Phe_ during exclusive breastfeeding (11.3‰ and 9.8‰) than the pre‐natal period (4.5‰), which we can tie to a maternal diet that included low‐quality protein sources. A change in MOM 1's diet during these phases, between 4 and 19 weeks, is evident by the increase in *δ* ^15^N_Phe_ and *δ* ^15^N_Glx_ of 6.5‰ and 2.6‰, respectively. MOM 1 also reported a lack of appetite during pregnancy, which only returned after she began breastfeeding. This may explain her higher TEF_Glx‐Phe_ during the prenatal period, with the enrichment further amplified by a low‐quality protein diet postpartum.

While TEF_Glx‐Phe_ for mother‐infant pairs seems indicative of dietary protein quality, it is crucial to recognize potential inaccuracies in diet interpretations arising from physiological or pathological effects on *δ* ^15^N_Phe_. Furthermore, the TEF_Glx‐Phe_ relies on the assumption that the Tr‐AA, glutamate, is enriched in ^15^N compared to the Src‐AA with each trophic transfer (Chikaraishi et al. 2009). However, negative TEF_Glx‐Phe_ values were observed in cases like MOM‐CHIL 2 during exclusive breastfeeding (Δ^15^N_Phe_ = 3.9‰; Δ^15^N_Glx_ = 2.7‰) and MOM‐CHIL 3 during the pre‐natal period (Δ^15^N_Phe_ = 1.3‰; Δ^15^N_Glx_ = 0.3‰), when *δ* ^15^N_Phe_ increased with trophic level or fluctuated due to physiological or pathological conditions (Table 2). This observation challenges the conventional assumption that *δ* ^15^N_Phe_ remains stable. Rather, our study suggests *δ* ^15^N_Phe_ is affected by physiological changes and thus influences CSIA interpretations derived from TEF_Glx‐Phe_.

## 5 Conclusions

This study highlights the effectiveness of CSIA‐AA in reconstructing breastfeeding and weaning practices. Specifically, the *δ* ^15^N of proline consistently demonstrated ^15^N enrichment and depletion associated with exclusive breastfeeding and weaning, supporting the findings of Harris et al. (2024). As proposed by Harris et al. (2024), we suggest that the trophic level effect of *δ* ^15^N_Pro_ is attributable to proline's role as a precursor in arginine synthesis. The combination of low arginine content and high proline content in breastmilk, coupled with the increased demand for arginine during growth, results in elevated arginine synthesis and subsequent proline catabolism. Although our sample size was small and two of the three mothers experienced pathological stressors, the consistent *δ* ^15^N_Pro_ patterning across all three dyads supports its reliability as a marker for tracking breastfeeding and weaning.

In addition to elucidating breastfeeding and weaning patterns, our CSIA‐AA results proved effective in identifying periods of physiological or pathological stress. Nitrogen isotope compositions of phenylalanine, and to a lesser degree, glycine, were the most indicative of stress. Glycine plays a crucial role as one of the primary substrates in gluconeogenesis, a metabolic pathway converting non‐carbohydrates into glucose, and glycine's subsequent deamination results in ^15^N enrichment during stressful periods. While the cause of enrichment in ^15^N for phenylalanine during stress is not fully understood, it may be linked to the distinctive turnover rates of Src‐AAs compared to Tr‐AAs. Further investigation into the catabolism of Src‐AAs in both infants and adults is necessary to effectively infer physiological, nutritional, or pathological stress from CSIA‐AA *δ* ^15^N data.

The impact of non‐dietary factors on *δ* ^15^N_Phe_ poses a significant challenge when interpreting the TP for both mothers and infants in this study, as well as for other bioarchaeological and ecological investigations. This challenge arises because much of the CSIA‐AA literature, including the equation established by Chikaraishi et al. (2009), assumes that phenylalanine is unaffected by non‐dietary factors; it is canonically considered a Src‐AA. Consequently, the application of indices based on that premise may lead to inaccurate interpretations. Moreover, the use of the TEF_Glx‐Phe_ in assessing dietary protein quality for infants is also compromised due to the influence of non‐dietary factors on *δ* ^15^N_Phe_. The current TEF_Glx‐Phe_ metric relies on the Tr‐AAs being enriched in ^15^N compared to the Src‐AAs for each trophic transfer. Therefore, we advise researchers studying the TP or TEF using *δ* ^15^N_Phe_ to exercise caution. We encourage further investigations that specifically focus on the effects of stress on *δ* ^15^N_Phe_ and phenylalanine metabolism, and which incorporate larger sample sizes and individuals with diverse dietary regimes.

Although contemporary infant feeding practices differ from those in archaeological populations, given the consistency of AA metabolism across human tissues, our findings support the use of CSIA‐AA for identifying weaning transitions in both modern and ancient contexts. In the absence of clinical or historical records, CSIA‐AA offers a valuable method for reconstructing individual health and nutrition. Future work should examine how health stressors, such as illness or injury, affect AA pathways—particularly phenylalanine—and expand applications across regions, age groups, and tissue types. These efforts will refine interpretations of diet, stress, and cultural context in past and present populations.

## Author Contributions

**Hana Salahuddin:** conceptualization, investigation, writing – original draft, methodology, writing – review and editing, formal analysis, data curation, funding acquisition, visualization. **Andrea L. Waters‐Rist:** funding acquisition, writing – review and editing, conceptualization, supervision. **Fred J. Longstaffe:** funding acquisition, supervision, writing – review and editing, validation, resources.

## Ethics Statement

This research adheres to the ethical guidelines outlined in the Tri‐Council Policy Statement on Ethical Conduct for Research Involving Humans (TCPS2) and the Ontario Personal Health Information Protection Act (PHIPA, 2004). The study protocol was approved by the Non‐Medical Research Ethics Board at Western University (Project ID 119889).

## Consent

All participants provided informed consent prior to their involvement in the study.

## Conflicts of Interest

The authors declare no conflicts of interest.

## Supporting information


**Data S1:** Supporting Information.

## Data Availability

Data that supports this study is available in the Supporting Information 1–5.
